# Bioimaging of alloantigen-stimulated regulatory T cells in rat vascularized composite allotransplantation

**DOI:** 10.1371/journal.pone.0203624

**Published:** 2018-09-07

**Authors:** Hui-Yun Cheng, Sheri K. L. Tay, Chih-Jen Wen, Chih-Fan Lin, Aline Yen-Ling Wang, Ling-Yi Shih, Shiao-Chin Liu, Eiji Kobayashi, Cheng-Hung Lin, Fu-Chan Wei

**Affiliations:** 1 Center for Vascularized Composite Allotransplantation, Linkou Chang Gung Memorial Hospital, Gueishan, Taoyuan, Taiwan; 2 Department of Plastic and Reconstructive Surgery, Linkou Chang Gung Memorial Hospital, Gueishan, Taoyuan, Taiwan; 3 Canniesburn Plastic Surgery Unit, Glasgow Royal Infirmary, Glasgow, United Kingdom; 4 Department of Organ Fabrication, Keio University School of Medicine, Tokyo, Japan; 5 School of Medicine, Chang Gung University, Gueishan, Taoyuan, Taiwan; University of Toledo, UNITED STATES

## Abstract

**Background:**

Tipping the balance toward regulatory T cells (Tregs) through adoptive cell therapy has shown promise to induce transplantation tolerance. Although such strategy has been explored in many mice organ transplantation studies, less knowledge was available in rat systems. Furthermore, the behaviors of the transferred cells have not been well studied in real-time fashion.

**Methods:**

Tregs from naïve LEW rats were purified in two steps with the autoMACS system. Immunosuppression potential of these cells was examined with mixed lymphocyte reaction. Following stimulation by the alloantigen *in vitro*, the purified Tregs were infused into the recipients of vascularized composite allotransplantation (VCA). Secondary allogeneic skin grafting challenge was performed on the recipients with long-term survived VCA. Live optical imaging was performed to track luciferase-expressing Tregs following infusion to the VCA recipients. Expression of relevant molecules was studied by flow cytometry or quantitative RT-PCR.

**Results:**

Rat Tregs were enriched following two-step cell sorting and showed immunosuppressive capacity. Upon infusion into the VCA recipients that have been treated with antilymphocyte serum and short-term Cyclosporin A, the antigen-stimulated Tregs significantly prolonged VCA survival and induced donor-specific tolerance. Tracking of the infused bioluminescent Tregs showed their specific homing to lymph nodes, and then to the VCAs. Following secondary skin grafting, Tregs specifically gathered at the donor-derived skin that was not rejected by the recipient. The *in vivo* migratory pattern coincided with the altered expression of cell surface molecules of CD62L, CD103, CD134, and CD278, following donor-antigen stimulation. Elevated expression of CCR4 and CCL22 in allograft may also participate in recruiting Tregs for maintenance of VCA survival and promoting donor-specific tolerance.

**Conclusion:**

Sorted Tregs induced donor-specific tolerance to VCA in rats. Live cell tracking demonstrated that activated CD4^+^CD25^+^FoxP3^+^ Tregs targeted primarily to the lymph nodes and VCA. The Tregs migrated to the secondary grafted donor skin and contributed to the maintenance of donor-specific tolerance. These behaviors were associated with phenotypic changes induced by donor antigen stimulation. Increased expression of CCR4 and CCL22 in VCA skin may also be relevant.

## Introduction

Vascularized composite allotransplantation (VCA) refers to the reconstruction of a recipient’s anatomical unit containing multiple tissue types, such as hand/forearm or face, by a corresponding part procured from a deceased donor [1, 2]. Since 1998, over one hundred patients have benefited from various kinds of VCA with impressive functional and aesthetic outcomes in most cases. However, wider application of VCA has been hindered by the requirement for lifelong non-specific immunosuppressants and the accompanying toxicities [3, 4]. Pursuing a donor-specific tolerance that allows complete withdrawal of immunosuppressants without harming allograft survival has therefore been the ultimate pursuit of transplant immunology [5, 6].

Donor-specific tolerance to VCAs has been accomplished by various approaches, such as bone marrow or adipocyte-derived stem cell therapy [7–9]. Although the mechanisms are yet to be fully characterized, current knowledge from organ transplantation and VCA has demonstrated the importance of CD4^+^CD25^+^FoxP3^+^ regulatory T cells (Tregs) for the induction and maintenance of tolerance to allotransplants. Elevated level of Tregs was observed in peripheral blood and VCAs in the recipients who had developed tolerance [7, 9]. Tregs were also detected in long-term tolerized islet, skin, renal, and cardiac allografts, and have been suggested to participate in maintaining tolerance (review in [10]). The presence of Tregs in the allograft has been associated with stable allograft function [11] whilst the depletion of Tregs inhibited donor-specific hyporesponsiveness [12].

Considerable efforts have been targeted towards developing Tregs as a cellular therapeutic agent. Adoptive transfer of Tregs to transplantation recipients may increase the ratio of Tregs to effector T cells, and provide a regulatory environment to promote tolerance. This strategy has been proven successful in prolonging allograft survival in animal models of organ transplantation as well as VCA [13–15]. Clinical trials on Treg-based therapy have demonstrated safety and potential to induce tolerance [16–18], although detailed mechanistic knowledge remains to be revealed.

In the current study, we demonstrated that adoptive transfer of antigen-stimulated CD4^+^CD25^+^ Tregs can prolong survival as well as induce donor-specific tolerance of rat VCA. Real-time tracking of infused luciferase-expressing Tregs showed these cells migrated to lymph nodes followed by VCA after infusion, and stayed in draining lymph nodes and VCA for the long-term. Furthermore, secondary skin grafting induced the migration of Tregs toward allograft skin, suggesting active recruitment of Tregs by the alloantigen is critical for maintenance of donor-specific tolerance. These behaviors were associated with phenotypic changes induced by donor antigen stimulation. CCR4 and CCL22 may participate in maintenance of Treg population in allografts and tolerance.

## Materials and methods

### Animals

Male 8–12 weeks old donor Brown-Norway (BN, RT1n) and recipient Lewis rats (LEW, RT1l), representing a full MHC mismatch, were purchased from the National Laboratory Animal Center, Taiwan. Luciferase transgenic LEW rats were provided by Professor Eiji Kobayashi at Keio University in Japan and bred in Chang Gung Memorial Hospital, Taiwan. All animals were housed in the animal facility of Chang Gung Memorial Hospital, under pyrogen-free conditions, with temperature and lighting cycles controlled, and water and commercial rat chow freely available. When applicable, the animals were anaesthetized with isoflurane, and euthanasia with carbon dioxide. All experiments were conducted in accordance with the Guide for the Care and Use of Laboratory Animals of the National Institutes of Health and following the Institutional Animal Care and Use Committee (IACUC) protocols authorized by Chang Gung Memorial Hospital, Taiwan, with the authorized protocol numbers of 2009121113, 2012121809, 2015032501, and 2016092601.

### Rat model of vascularized composite allotransplantation

A previously described heterotopic hindlimb osteomyocutaneous VCA model was used [7]. Briefly, VCA harvest in the isoflurane-anaesthetized donor rat began with a longitudinal medial hindlimb incision from ankle to groin; this was extended to delineate the skin paddle (4 cm x 3 cm). Proximal to the ankle the tendons were cut, tibial vessels cauterized and the tibia osteotomized. The superficial epigastric vessels were ligated, thigh muscles sectioned and femur osteotomized. The flap was isolated on the femoral vessels below the inguinal ligament. The flap, including medullary cavities, was flushed with 10 ml heparinized saline, wrapped in saline gauze and placed on iced saline. The donor rat was euthanized with CO_2_. In recipient rats, 3 cm inguinal and gluteal skin incisions were performed to prepare the recipient femoral vessels and delineate the recipient defect, respectively. The VCA was then inset and its circulation restored by 10/0 nylon microanastomoses. The inguinal incision was closed and the animal recovered. VCAs were then evaluated daily with an established semi-quantitative rejection grading system that ranges in severity from grade 0 to 4 as follows: grade 0, no rejection; grade 1, pink or slightly erythematous; grade 2, frank erythema; grade 3, erythema or purplish discoloration with blister formation or partial hair loss; and grade 4, dark purplish discoloration with blister formation and major hair loss. Rejection was defined when 80% of the VCA reached grade 4. Histological changes and lymphocyte infiltration were evaluated by microscopy after hematoxylin and eosin (H&E) staining.

### VCA study groups

A total of 29 heterotopic hindlimb osteomyocutaneous flaps were transplanted in 8–12 week-old male rats and divided into 4 groups. The recipient LEW rats received syngeneic transplants (group 1) or allogeneic transplants (groups 2–4) from BN rats on day 0. Groups 1 and 2 did not receive additional treatment. Groups 3 and 4 were treated with 0.5 ml ALS intraperitoneally on day -1 and +2, and daily subcutaneous CsA (16 mg/kg) from day 0 to 7. On POD 10, group 4 recipients were infused intravenously with LEW Tregs, which had been co-cultured with irradiated BN splenocytes for 72 hours.

### Skin grafting

When the VCA survived for 120 days, secondary skin grafting was performed as described in detail previously [7]. Briefly, dorsal cutaneous defects were created in recipients with long-term surviving VCA for insetting BN, and SD-origin full-thickness tail skin grafts (2 cm x 1 cm). These were fixed with tie-overs for 7 days and successful grafts evaluated daily for rejection for another 60 days. Rejection was evidenced by erythema, edema, scaling of the skin, hair loss, epidermolysis, and desquamation; destruction of more than 80% of the graft defined rejection.

### Isolation of CD4^+^CD25^+^ Treg cells

The spleen from a naïve LEW rat was harvested through a 1 cm left subcostal incision. It was then gently mashed in serum-free RPMI 1640 and filtered through a nylon mesh. Following lysis of erythrocytes with ACK buffer, cells were re-suspended in HBSS and counted. Sorting was performed using autoMACS Pro system (Miltenyi Biotec, Bergisch Gladbach, Germany) according to the manufacturer’s instructions. Briefly, splenocytes were re-suspended in MACS solution to a concentration of 10^8^ cells/ml and stained with antibodies for CD90.1, CD8a, CD11b/c, CD45RA and NKR-P1A (all were purchased from BD Pharmingen, San Jose, CA, USA) for 15 minutes at 4 °C, washed and followed by incubation with anti-IgG microbeads for 15 min at 4 °C. The CD4^+^ lymphocytes were collected through negative selection with the autoMACS Pro running program Possel. The CD4^+^-enriched fraction was then incubated with phycoerythrin (PE)-conjugated anti-CD25 (BD Bioscience, San Diego, CA, USA). The cells were then mixed well with MultiSort stop reagent and incubated with 20ul Anti-PE MicroBeads per 10^7^ cells for 15min at 4 °C. Following washing, CD4^+^CD25^+^ cells were collected with the autoMACS Pro by positive selection using program Possel. Isolated CD4^+^CD25^+^ Tregs were incubated with BN antigen-presenting cells at 1:1 ratio for 3 days at 37 °C in the presence of rat IL-2 (Peprotech, Rehovot, Israel). The cells were collected, washed and re-suspended in PBS before infusion. Due to the variation derived from sorting efficiency and cell culture, the total cell numbers for infusion were hard to be fixed, and were between 7x10^5^-2x10^6^.

### Flow cytometric analysis of sorted Treg cells

Expression of CD62L, CD103, CD134 and CD278 of sorted cells were analyzed by flow cytometry. Briefly, CD4^+^CD25^+^-enriched cells right after sorting or after donor antigen stimulation for 3 days were incubated with FITC-conjugated anti-CD62L (AbD Serotec, Kidlington, UK), APC-conjugated anti-CD103 (BioLegend, San Diego, CA, USA), FITC-conjugated anti-CD134 (AbD Serotec), or PE-Cy7-conjugated anti-CD278 (Biolegend) in addition to anti-CD4 (APC or PE-Cy7 conjugated) (BD Pharmigen). Followed by treatment with permeabilization kit (eBioscience) at 4 °C for 18 hr and staining with PerCP-conjugated anti-FoxP3 (eBioscience), the cells were analyzed by FACSCanto II flow cytometer (BD Biosciences). For analysis, live cells excluding debris and dead cells were gated first followed by gating on the FoxP3-expressing cell. Expression level of CD62L, CD103, CD134 and CD278 was analyzed on FoxP3-expressing cells specifically.

### Mixed lymphocyte reaction (MLR)

Rat spleens were harvested in sterile conditions. After erythrocyte lysis, splenocytes were isolated and re-suspended in complete RPMI-1640 media. In one-way MLR, LEW splenocytes (1x10^5^ cells/well) were co-cultured with either 2 μg/ml Concanavalin A (Con A, Sigma-Aldrich, St. Louis, MO USA) or allogeneic BN stimulator splenocytes that had been irradiated (2000 cGy; Gammacell^®^ 1000 Elite Nordion International, Ottawa, ON, Canada). Tregs were added in 1:1, 1:0.5, 1:0.2, 1:0.1 ratios to responder cells according to experimental design as described. Cells were cultured in quadruplicate in 96-well U-shaped plates for 4 days then pulsed with 1 μCi/well ^3^H-thymidine (^3^H-TdR, Perkin Elmer, Waltham, MA, USA) for 16 h and harvested over glass fiber filters. Thymidine uptake was quantified on a microplate scintillation and luminescence counter (Packard NXT, Meriden, CT, USA). Thymidine incorporation into spontaneously proliferating responders (in media alone) was the control and set as 1. Ratios of thymidine incorporation under all other conditions with respect to the control were acquired, providing stimulation indices (SI).

### *In vivo* dynamic cell tracking with IVIS Spectrum

CD4^+^CD25^+^ Tregs were isolated from luciferase transgenic LEW rats as described earlier. Following infusion to VCA recipients, luciferase-expressing Tregs were tracked *in vivo* with IVIS Spectrum (Xenogen, Alemeda, CA, USA) at pre-designated intervals till sacrifice.

### RNA preparation and quantitative RT-PCR

Skin tissue was preserved in RNAlater (Thermo Fisher, Waltham, MA, USA) and minced under liquid nitrogen followed by dounce homogenization in TRIZOL^®^ Reagent (Invitrogen, Carlsbad, CA, USA) according to manufacturer’s instructions. Concentration of the collected DNA is measured by OD_260_. Only the RNA with a OD_260_/ OD_280_ ratio higher than 1.8 proceeded to be studied further. Each RT-PCR is derived from 10 ng of total RNA isolated. For reverse transcription, 10 ng of total RNA was incubated with dNTP mix, primer and Superscript II Reverse Transcriptase (Invitrogen) under 16°C for 30 min, followed by 42°C for 30 min and 85°C for 5 min in a total volume of 15 ul. The PCR reaction was done in a total reaction volume of 20 μl, containing 100 ng cDNA, 10 pmol each of the forward and reverse primers, 10 μl SYBR Green PCR master mix (Applied Biosystems, Foster City, CA, USA). The mixture was preheated at 95 °C for 10 minutes and then cycled for 40 times at 95 °C for 1 minute for denaturation and 60 °C for 1 minute for annealing and elongation in a ABI StepOne system (Applied Biosystems). The progress of the fluorescence generation by chelation of SYBR Green to the double-strand PCR product was continuously monitored. The threshold cycle (Ct) for each reaction was acquired for quantitation with GAPDH serving as the internal control.

### Statistics

Data were expressed as mean ± SD unless otherwise indicated. Results of MLR were analyzed by one-way ANOVA followed by Tukey-HSD for *post hoc* pairwise comparison. Results of flow cytometry were analyzed by Student’s t test. Median VCA survivals with standard errors were acquired by the product limit method of Kaplan-Meier and presented by survival curves. A probability value less than 0.05 was regarded as statistically significant. All statistical analyses were conducted with SPSS software.

## Results and discussion

### Immunosuppressive Tregs can be isolated with MACS system

**Fig 1:**
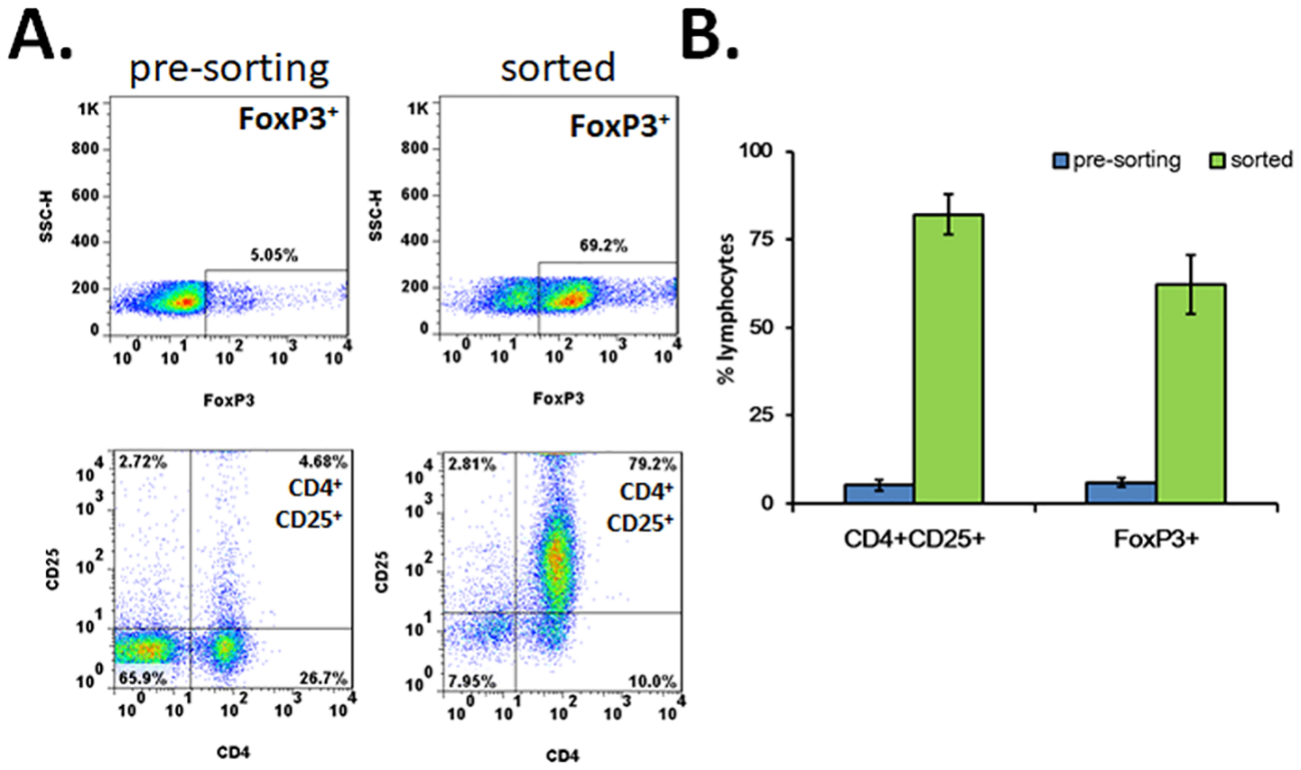
Sorting with autoMACS Pro enhanced the purity of CD4^+^CD25^+^FoxP3^+^ Tregs. A. Representative flow cytometric diagrams from splenocytes before (left column) and after (right column) sorting. The percentages of cells expressing FoxP3 (upper panel) and CD4^+^CD25^+^ in lymphocytes are shown. B. Group data on the purity of CD4^+^CD25^+^ and FoxP3^+^ cells in lymphocytes before and after sorting.

We isolated Rat Tregs from the spleens of LEW rats in two steps with magnetic-activated cell sorting (MACS) technology. The first step was negative selection to deplete CD90.1^+^, CD8^+^, CD11b/c^+^, CD45RA^+^ and NKR-P1A^+^ cells and enrich CD4-expressing cells. Following positive selection of CD4^+^ cells with CD25 expression, the purity of CD4^+^CD25^+^ cells increased from 4.68% to 79.2% on average. The percentage of FoxP3-expressing cells increased from 5.05% to 69.2% (Fig 1). We also tried to isolate CD4-expressing cells through positive selection at the first step. However, the yield of CD4^+^CD25^+^ cells was very low (data not shown). It could be possibly due to the interference of CD25 antibody binding by the CD4 antibody-bead complex bound to the cells at the first sorting step.

**Fig 2:**
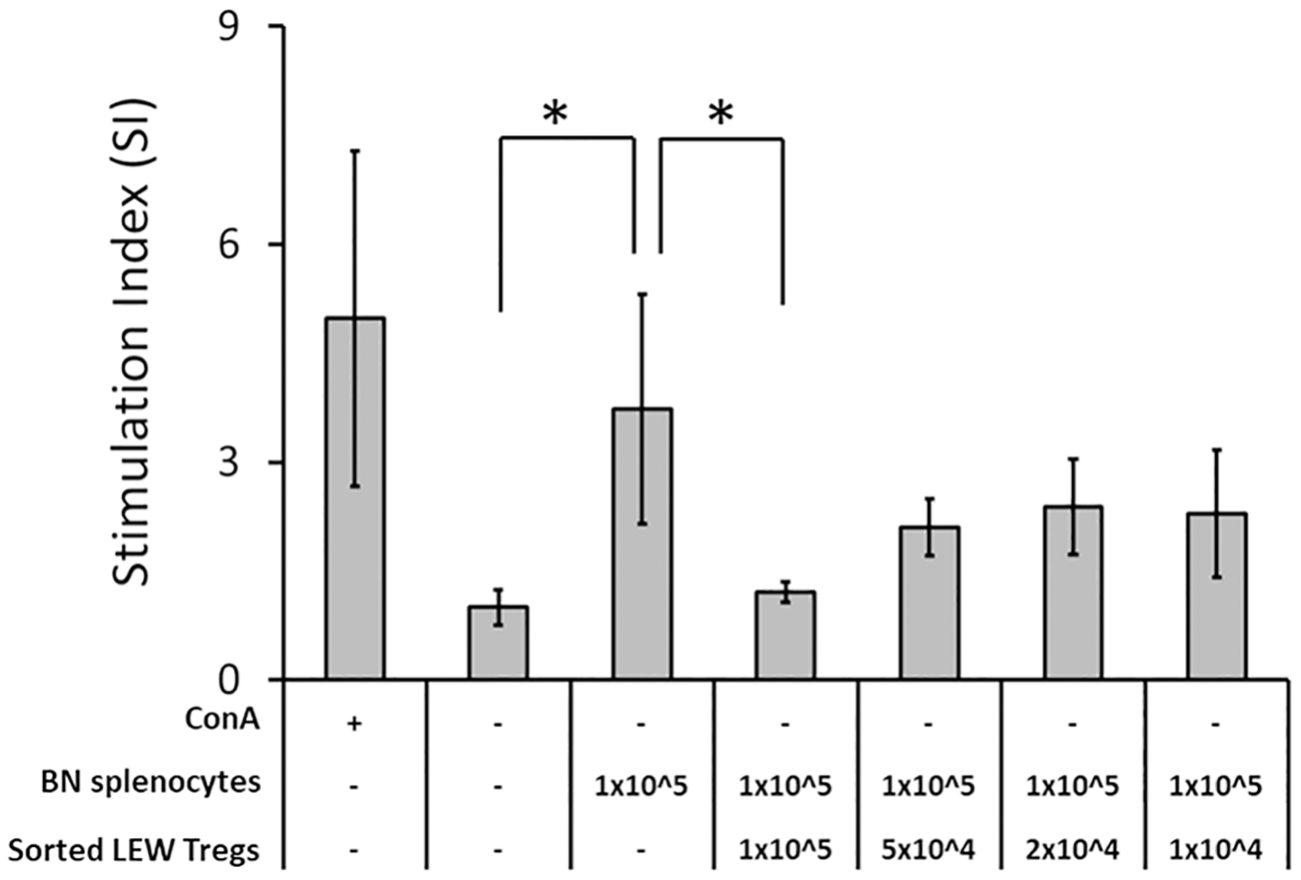
Sorted Tregs suppressed BN splenocyte-induced proliferation of LEW splenocytes in one-way MLR. Each reaction contained 1x10^5^ LEW splenocytes. Mitogen Con A, BN splenocytes or sorted LEW Tregs were added in reaction as indicated. Cellular proliferations were expressed as mean stimulation indices (SIs) with respect to spontaneous proliferation of four experimental repeats with standard deviation. The asterisk denotes statistical significance acquired with ANOVA followed by post hoc Tukey’s test with the probability less than 0.05.

The immunosuppressive functions of these CD4^+^CD25^+^-enriched cells were evaluated by mixed lymphocyte reaction (MLR), in which LEW splenocytes served as responders and irradiated BN splenocytes as allogeneic stimulators. Compared to spontaneous proliferation where LEW splenocytes were cultured in media alone, a significant increase in LEW splenocyte proliferation was observed when they were co-cultured with stimulators or mitogenic Con A. Adding sorted CD4^+^CD25^+^ cells suppressed proliferation of the responder in a dose-related manner against BN antigen stimulation. When added at an equivalent cell ratio (ie. LEW: Treg ratio of 1:1), BN antigen-induced proliferation of responder cells was almost completely suppressed to the level of spontaneous proliferation. Less suppression in responder proliferation was detected when fewer Tregs were added (Fig 2).

### Adoptive transfer of donor antigen-stimulated Tregs prolonged survival of vascularized composite allotransplants

Tregs were known to exert a suppressive function through secretion of inhibitory cytokines, cytolysis and metabolic interruption of effector cells, as well as modulation of dendritic cells [19]. The adoptive transfer of Tregs has been shown to prolong allograft survival in several different systems. Furthermore, Tregs with donor-specificity were shown to have better suppressive function against donor antigen-elicited alloresponse both *in vitro* and *in vivo* [20]. Therefore, we stimulated the isolated Tregs with donor antigens, followed by adoptive transfer to the VCA recipients, and then evaluated the VCA survival.

**Fig 3:**
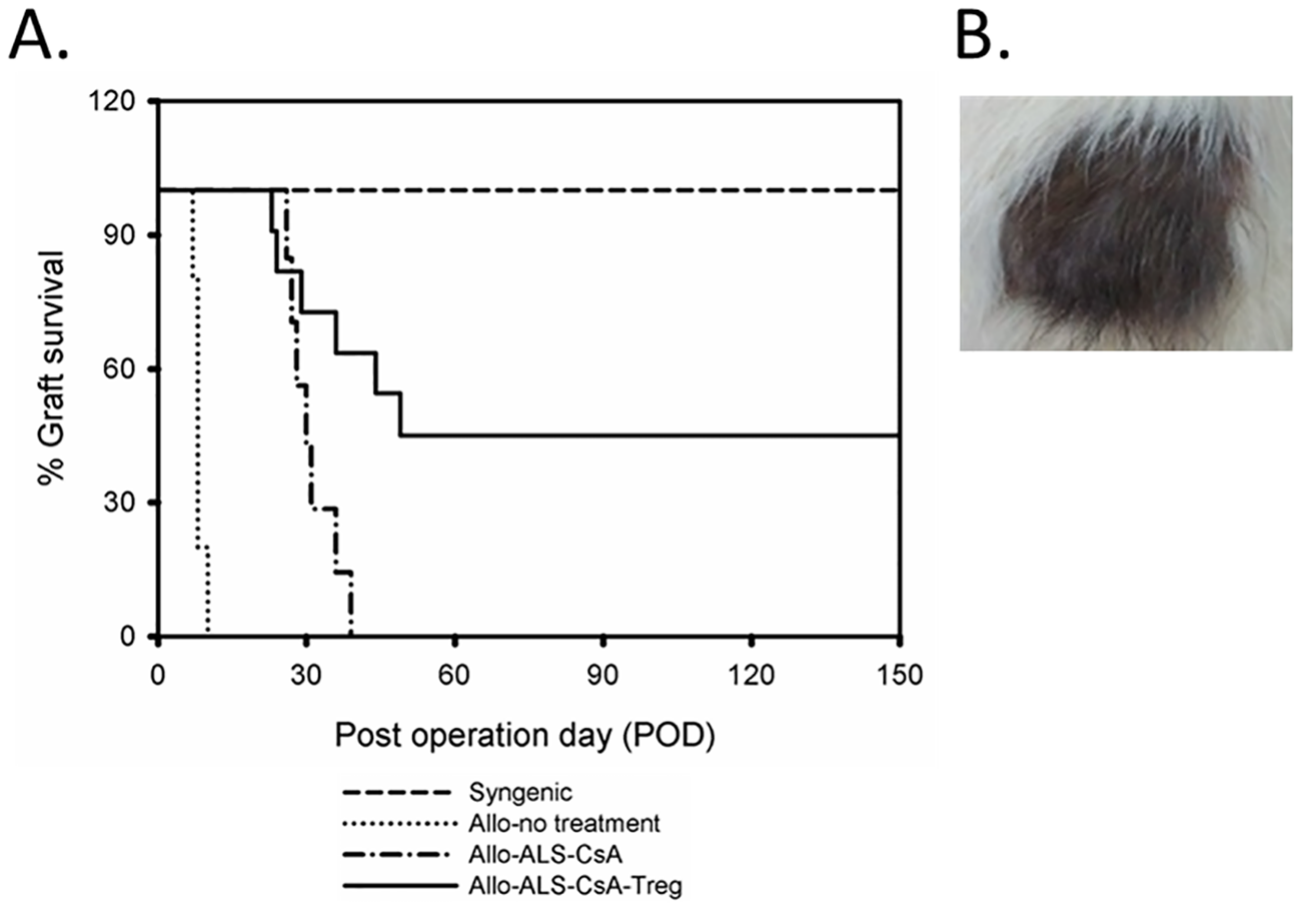
Infusion of BN antigen-stimulated Tregs significantly prolonged VCA survival. A. Kaplan-Meier survival curves for control and experimental groups. B. Outlook of VCA after long-term survival at POD 154.

All syngeneic transplants survived the whole observation period of 150 days without signs of rejection (n = 6), whereas group 2 (rejection control, n = 5) animals rejected VCAs between days 7 and 10. Groups 3 (n = 7) and 4 (n = 11) showed significant prolongation of VCA survival compared to group 2. Sole treatment with immunosuppressants (ALS and CsA) moderately prolonged graft survival to around POD 30. Infused Tregs significantly increased the ratio of VCA recipients that had long-term VCA survival (to POD 150) to 45%, with group median survival time of 87 days (Fig 3).

### *In vivo* cell tracking demonstrated that adoptive transferred Tregs migrated to and homed towards lymph nodes and allograft and persisted at these sites long term

**Fig 4:**
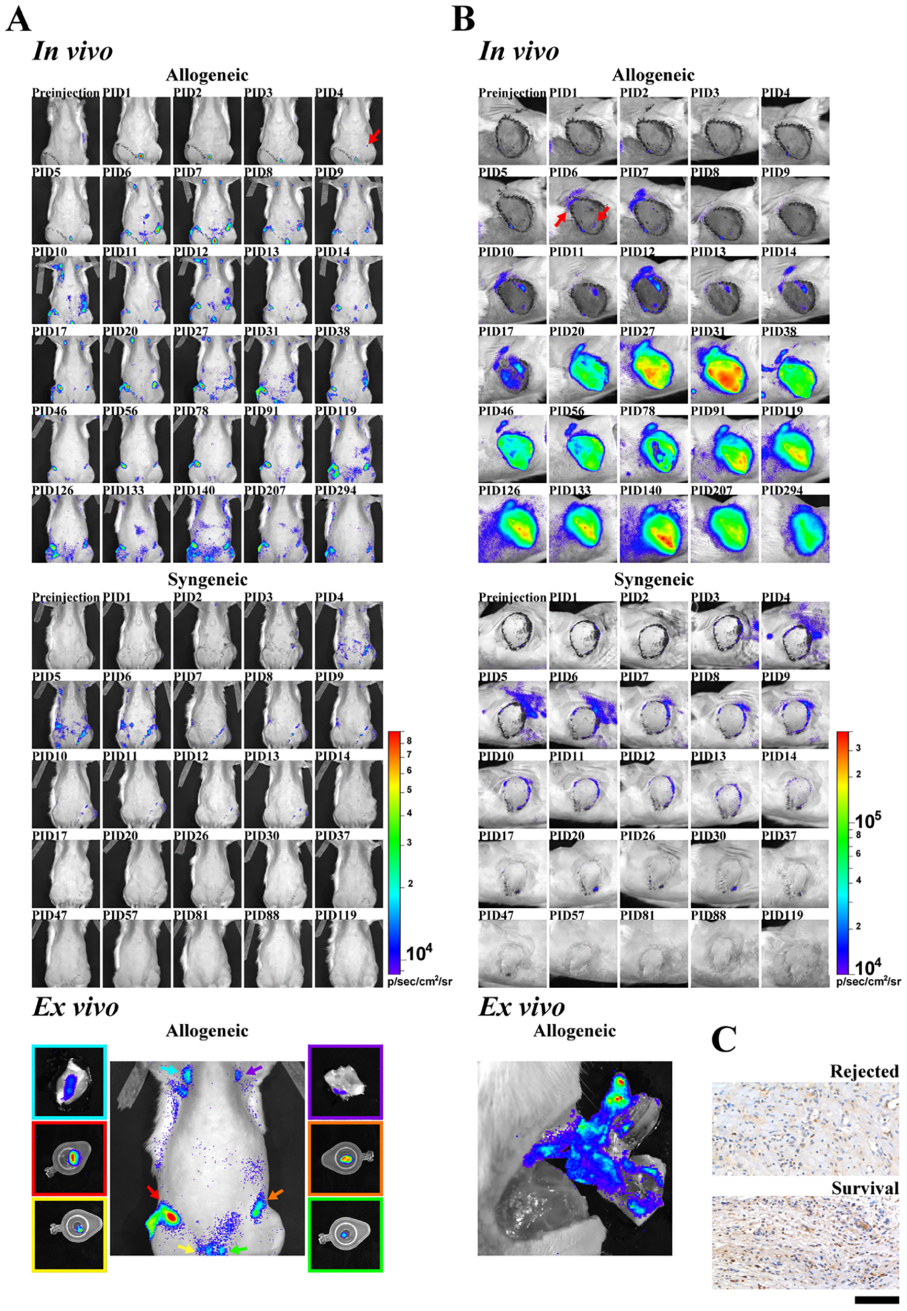
Bioluminescent imaging of luciferase-expressing Tregs following infusion. The upper and middle panels show the traces of infused Tregs in recipients of allograft (upper) and syngeneic graft (middle). The bottom panel demonstrates the *ex vivo* validation following dissection of the allogeneic recipient at PID 294. Photo frames of the tissues and the dissection sites were color-matched. A. ventral view. B. transplanted graft. In the recipient of allogeneic graft, infused Tregs migrated to and stayed at the lymph node(s) from PID 4, whereas they appeared at the allograft from PID 6 (shown with red arrow) and intensified by PID 20. The signal was maintained throughout the whole observation period. In comparison, no clear trace of infused Tregs was observed in syngeneic recipients after two weeks. PID: post Treg-infusion day. PID 1 = POD 11. (C). Rejected and tolerant VCAs were stained with FoxP3 antibody followed by counterstaining with hematoxylin. FoxP3-expressing cells were characterized by brown staining, whereas blue staining were derived from cell nuclei. Scale bar represents 100μm.

The behaviors of the infused cells *in vivo* were tracked by bioluminescence imaging with Tregs isolated from LEW rats with luciferase transgene expression [21] in the presence of luciferin. This approach also allowed us to specifically monitor the behaviors of the infused cells. As shown in Fig 4, infused Tregs migrated to the inguinal lymph node at as early as 4 days after infusion (PID 4, equals to POD 14), and the axillary lymph nodes at PID 6 in the allograft recipients. The signal remained for long term, although that of the axillary lymph nodes lessened after long-term tracking. Furthermore, intense bioluminescence was observed at the VCA from PID 17 and this level was stable and persisted for as long as the allograft survived (Fig 4, upper panel). The bioluminescence signals were confirmed to be coming from lymph nodes and allograft following dissection (Fig 4, lower panel). On the other hand, no clear migration pattern was observed in the recipient of syngeneic graft infused with the same number of luciferase-expressing Tregs. The luciferase signal faded away after two weeks (Fig 4, middle panel). It suggests that the allogeneic environment was critical for maintaining the infused Treg population, similar to the finding reported in a mice graft versus host disease model [22].

**Fig 5:**
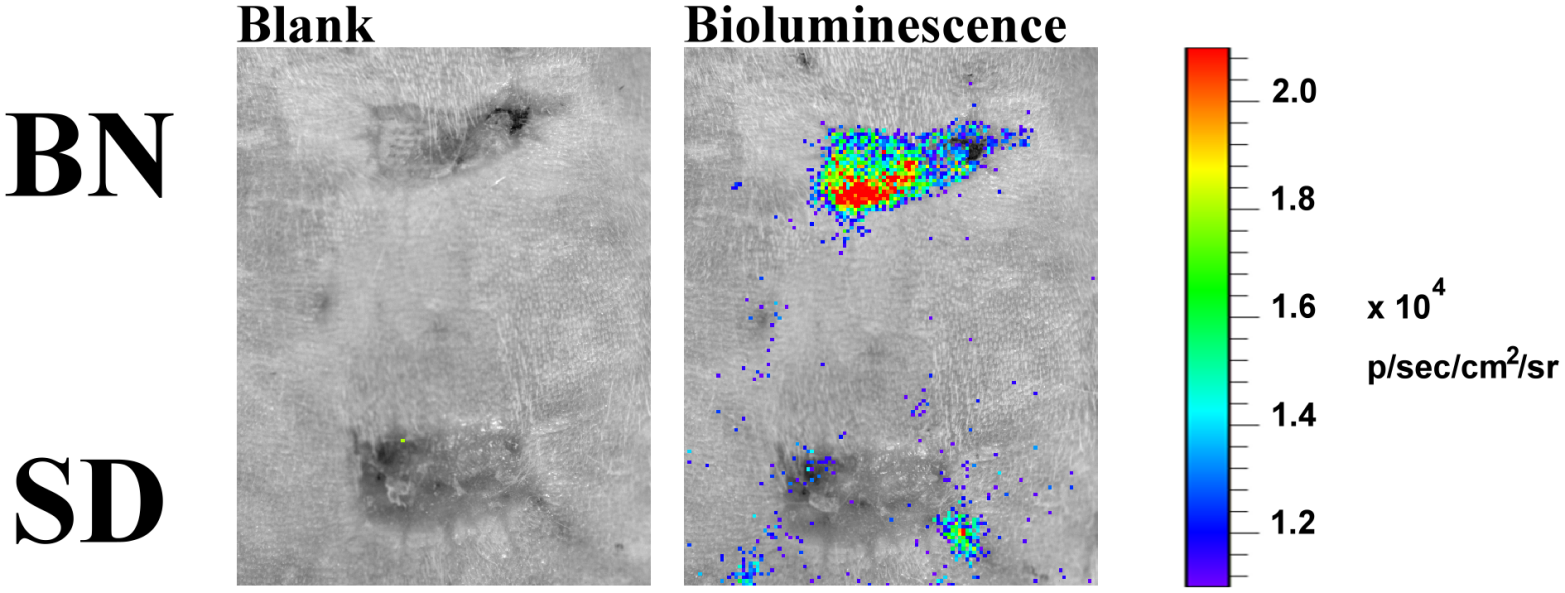
Bioluminescent imaging of luciferase-expressing Tregs following secondary skin grafting. Blank (without luciferin) and bioluminescence images (with luciferin) of the skin grafts derived from the donor strain (BN) or the third-party strain (SD) were taken at 60 days after skin grafting.

Recipients with long-term survived VCAs were challenged with secondary skin grafting derived from the original donor strain BN or a third-party strain SD. The SD skin was rejected and a scar following secondary healing was observed. In comparison, the BN skin survived well, suggesting the donor-specific tolerance was established. Interestingly, the luciferase-expressing Tregs infused early after VCA were found to migrate and concentrate specifically to the BN skin, whilst there was no bioluminescence detected in the third-party graft (Fig 5). Consistent with the finding, immunohistochemical study of FoxP3 also showed that Tregs were concentrated in the tolerant VCA and were absent in the rejected skin (Fig 4C).

However, Zhang et al previously demonstrated in a mouse islet allotransplantation model that infused Tregs first migrated to the allograft, become activated, and subsequently migrated to draining lymph nodes [23]. Later studies identified that T-bet and lymphotoxin beta receptors participate in afferent lymphatic migration in this system [24, 25]. The authors suggested such migration pattern was critical for optimal suppression of alloimmune response [23]. Nevertheless, this study was performed in a cross-sectional fashion with microscopic observation on samples collected at designated time points and then the sequence of events was reconstructed. In contrast, real-time *in vivo* imaging was performed in our study, and the behavior of Tregs was observed longitudinally in live animals. Our observation showed a different migration pattern with the Tregs homing first to the lymph nodes before migrating to the allograft. One factor that may explain the observed discrepancy is the timing of the Treg infusion. Zhang et al transferred Tregs at the time of transplantation, whereas we conditioned the recipients with ALS and short-term cyclosporine for one week till three days prior to Treg infusion. At the time when Tregs enter the recipient system, donor antigens may have already been processed to prime recipient T cells at the lymph nodes. Thus the infused Tregs preferentially migrated to the lymph nodes to prevent T cell priming and expansion of Teffs which specifically reacted to donor antigens. Our finding is consistent with the observation by Nguyen et al with *in vivo* cell tracking that infused Tregs migrated to LNs and proliferated prior to moving to peripheral tissues. Their imaging study also demonstrated that proliferation of conventional CD4^+^ and CD8^+^ T cells was suppressed in the presence of Tregs [22].

### Donor antigen stimulation induced phenotypic changes on isolated Tregs

**Fig 6:**
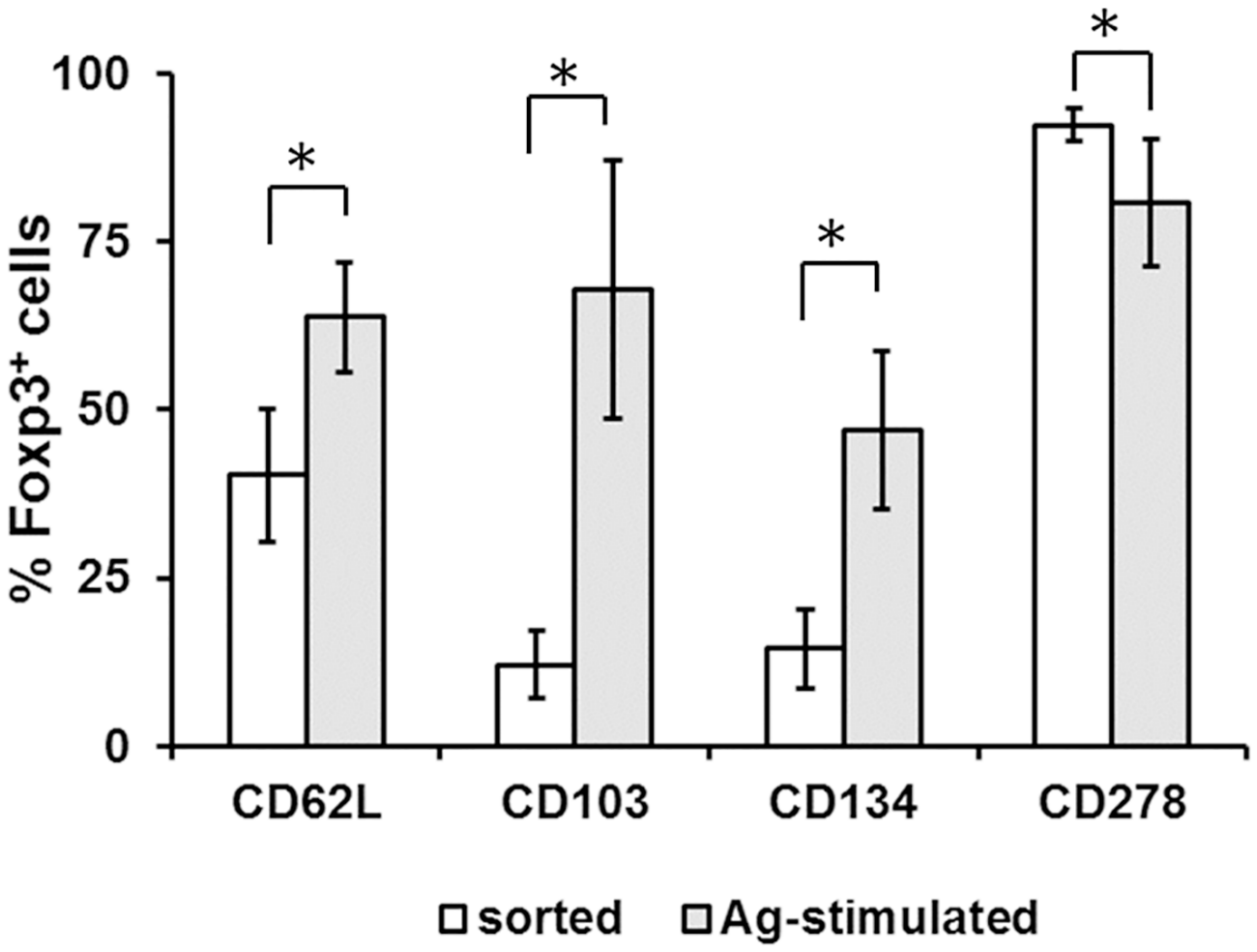
*In vitro* stimulation with donor (BN) antigens induced phenotypic changes of sorted Tregs. BN antigen stimulation induced significantly higher expression of CD62L, CD103, and CD134 on sorted Tregs. By contrast, lower expression of CD278 was observed. The asterisk denotes statistical significance acquired with Student’s t test with the probability less than 0.05.

Since the donor antigen stimulated Tregs significantly prolonged VCA survival and induced donor-specific tolerance, how donor antigen stimulation affected the cell surface expression of CD62L, CD103, CD134, and CD278 on sorted Tregs were studied *in vitro* by flow cytometry. As shown in Fig 6, expression of the surface molecules CD62L, CD103, and CD134 on FoxP3^+^-expressing Tregs were significantly increased. By contrast, slightly lower levels of CD278 were observed on alloantigen-stimulated Tregs with statistical significance.

Tregs have been demonstrated to be versatile and consistently respond to the immune environment with subtle modifications in phenotype and function in the periphery [26]. In line with this, we found that donor antigen stimulation increased the surface expressions of CD62L and CD103. CD62L, also known as L-selectin, has been well studied as a homing signal for lymph nodes [27]. Furthermore, CD62L^+^ T cells were more responsive to alloantigen stimulation compared to the CD62L^-^ counterparts [28]. Our data of enhanced expression of the LN-homing CD62L on Tregs by donor antigen stimulation, further supported the preferential LN-targeting behavior of the infused Tregs that we observed (Fig 4). CD103, encoding the α_E_ chain of α_E_β_7_ integrin, was shown to participate in the retention of Tregs at infection sites [29], as well as regulation of the suppressive function of Tregs [30]. In a murine GVHD model, CD103^+^ Tregs were shown to induce apoptosis of Teff and B cells at the target site [31].

CD134 (OX40), and CD278 (ICOS) are both co-stimulatory molecules for T cells [32, 33]. Consistent with our findings, Miura reported that alloantigen stimulation significantly upregulated expression of CD134 and FoxP3 on CD4 lymphocytes [34]. CD134 was shown to regulate Treg function since administration of CD134 antibody abrogated suppression mediated by Tregs in a GvHD model, in addition to its participation in development and homeostasis of Tregs [35]. Expression of CD278 was shown to define subsets of Tregs with differences in cytokine production [36], as well as viability and suppressive capability [37]. In the current study, donor antigen stimulation induced a small but significant decrease in CD278 expression on Tregs, suggesting a less homogeneous Treg population was generated with antigen stimulation. Our data suggest the alloantigen-specific Tregs may induce donor-specific tolerance through regulation of surface molecule expression. These Tregs participate in maintaining VCA survival through specific migration to lymph nodes then to the allografts, potentially inducing apoptosis of Teff, interfering with T cell infiltration, or preventing the generation of alloreactive memory CD8^+^ T cells locally [38, 39].

### Specific chemokines helped to recruit Tregs to the allografts

**Fig 7:**
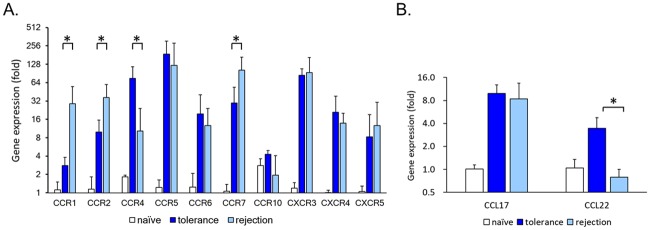
Higher expression levels of CCR4 and CCL22 in tolerant VCA skin than the rejected ones. A. group data showed relative expression levels of CCR1, 2, 4, 5, 6, 7, 10, and CXCR3, 4, 5 on naïve, tolerant, and rejection VCA skins. Only CCR4 expressed significantly higher level in tolerant versus rejected VCA skin. B. group data showed the CCR4 ligand CCL22 expressed significantly higher level in tolerant versus rejected VCA skin. Transcripts were RT-PCR amplified with gene-specific primers incorporating SyBr Green. The Ct (threshold cycle) was acquired and normalized to that of GAPDH. The average level of the naïve group was set as 1. The average with standard deviation of each group is shown. Asterisk denotes statistical significance between tolerant and rejection groups.

Recruitment of Tregs may be regulated by the interaction between chemokine and chemokine receptors as well [40]. Since the infused Tregs migrated to and then stayed at the tolerant VCA for extended period of time, gene expression of various chemokine receptors that may participate in lymphocyte recruitment in VCA was evaluated. We performed quantitative RT-PCR and compared the expression levels of CCR1, 2, 4, 5, 6, 7, 10, and CXCR3, 4, 5 on naïve (BN skins collected before surgery), tolerant and rejection VCA skins (Fig 7A). All chemokine receptors were upregulated in both tolerant and rejection skins. Post-hoc pairwise comparisons between tolerant and rejected VCA skins showed that CCR4 expressed significantly higher level in tolerant skins, whereas CCR1, 2, and 7 showed opposite expression pattern. The potential CCR4 ligands, CCL17 and CCL22, were then examined, and the data showed that CCL22 was upregulated significantly in tolerant VCA skins although CCL17 was not differentially expressed between the tolerant versus rejection skins (Fig 7B). It is thus reasonable to hypothesize that CCR4-CCL22 axis participates in recruiting/maintaining Tregs at the VCA skins to suppress alloimmune response locally. Our data along with that reported by Lee et al showing the requirement of CCR4 and CCL22 expression for recruitment of Tregs to the allogeneic cardiac transplant [41], support the significance of CCR4-CCL22 and Tregs in maintenance of allograft survival at both solid organ and composite tissue allotransplantation.

Since all other chemokine receptors were upregulated in tolerant skin compared to the naïve ones, their roles in recruiting Tregs for tolerance induction are not clear. Further analysis of the evolution of chemokine receptor panel on Tregs following donor antigen stimulation, or during retention at lymph nodes, may help to further delineate the fine molecular cues regulating Treg migratory behaviors.

## Conclusion

Our study demonstrates that rat CD4^+^CD25^+^ Tregs could be enriched with a two-step MACS protocol. Following stimulation by donor antigen, the sorted Tregs went through phenotypic changes *in vitro* and promoted allograft survival *in vivo*. Bioluminesence tracking of the infused Tregs in the wild type VCA recipient showed that these cells displayed a specific homing to lymph nodes and to the VCA. The signal sustained for long term in the recipient that developed donor-specific tolerance. The infused Tregs specifically migrated to donor rather than the third-party skin following a secondary antigen challenge by skin grafting, suggestive of an active and protective role of Tregs in long-term maintenance of VCA survival.
